# Urethrovaginal fistula following vaginal prolapse of a pedunculated uterine myoma: a case report

**DOI:** 10.1186/s13256-017-1457-2

**Published:** 2017-10-21

**Authors:** Elie Nkwabong, Joseph Nelson Fomulu

**Affiliations:** 1 Department of Obstetrics and Gynecology, University Teaching Hospital/Faculty of Medicine and Biomedical Sciences, P.O. Box 1364, Yaoundé, Cameroon; 2 Department of Obstetrics and Gynecology, University Teaching Hospital, Yaoundé, Cameroon

**Keywords:** Prolapsed submucosal uterine fibroid, Puerperium, Urethrovaginal fistula

## Abstract

**Background:**

Urethrovaginal fistulas are usually secondary to a foreign body in the vagina or to vaginal gynecologic surgeries. We present a case of an urethrovaginal fistula secondary to vaginal prolapse of a huge pedunculated submucosal uterine myoma.

**Case presentation:**

A 25-year-old black African woman with a past history of huge uterine fibroids and an uncomplicated vaginal delivery 5 weeks prior to presentation consulted for a difficult micturition that occurred 2 days earlier. A vaginally prolapsed huge uterine myoma was diagnosed. The fibroid was easily twisted off per vagina. Around 9 days after prolapse of the fibroid or 5 days after its removal, she complained of a vaginal leaking of urine during micturition. An urethrovaginal fistula was diagnosed using a blue dye test. The fistula was successfully repaired with polyglactin and she was discharged on day 15.

**Conclusions:**

To the best of our knowledge, this is the first case of urethrovaginal fistula secondary to delivered uterine myoma. We recommend close postpartum follow-up of women carrying huge uterine fibroid and urgent management of a vaginally prolapsed uterine fibroid to reduce the risk of urethrovaginal fistula.

## Background

Obstetrical fistula is usually the consequence of obstructed labor, with vesicovaginal and rectovaginal fistulas being the most frequent. These fistulas are secondary to bladder or rectal ischemia secondary to compression of these organs by the fetal presenting part, followed by necrosis and sloughing of the ischemic tissue, thereby, creating a communication between the vagina and the bladder or the rectum. Urethrovaginal fistula is another entity that is usually secondary to a vaginal foreign body [1, 2] or to gynecologic surgery [3]. No case of urethrovaginal fistula due to delivered myoma has been published. We present a case of an urethrovaginal fistula that followed a vaginal prolapse of a pedunculated uterine myoma.

## Case presentation

A 25-year-old black African woman, gravida 1 para 1, presented on the 15 May 2015 for difficult micturition that occurred 2 days earlier, followed by urinary retention. She had a term vaginal delivery 5 weeks earlier of a baby girl who weighed 3000 g. She had known intramural uterine fibroids confirmed by ultrasound scan 3 years earlier. On physical examination, her lower abdomen was distended. On speculum and digital vaginal examinations, a foul-smelling mass of approximately 10 cm in diameter was present in her vagina. The diagnosis of urinary retention due to a superinfected prolapsed pedunculated uterine fibroid was made. An indwelling plastic urinary catheter was easily placed. Antibiotic therapy was then started intravenously with ceftriaxone (1 g twice daily) and metronidazole (500 mg thrice daily). Two days later, under general anesthesia, the almost necrotic fibroid was held with two big toothed forceps and easily twisted off per vaginal route, without any significant bleeding. A speculum examination found a normal cervix, which was 4 cm dilated and the base of the fibroid’s pedicle was located in the posterior uterine wall at 3 cm from the external cervical os. The fibroid was sent for pathology, which later confirmed uterine leiomyoma. Antibiotics were continued for another 6 days. The catheter was removed 4 days after its placement, but 3 days after the removal of the catheter (around 9 days after prolapse of the fibroid or 5 days after fibroid removal) our patient started complaining of urine coming partly from her urethral meatus and partly from her vagina during micturition. Under general anesthesia, an urethrovaginal fistula of 3 mm diameter located 3 cm from the urethral meatus was diagnosed using the blue dye test. The fistula was successfully closed in two layers (urethral wall and vaginal wall) with polyglactin. A new catheter was placed and kept for 14 days. Her postoperative period was uneventful and she was discharged on day 15.

## Discussion

Fistulas involving the urethra are rare and are observed usually with an intravaginal foreign body [1, 2] or gynecologic surgery [3]. The foreign body compresses the vaginal and urethral walls, leading to ischemia and sloughing of vaginal and urethral tissue with fistula formation as the consequence. This has been observed in women with a hard-plastic intravaginal foreign body [1, 2]. Moreover, during gynecologic surgery, the urethra can be traumatized with a needle, a surgical blade, or another instrument [3].

Urethrovaginal fistula can be due to a vaginally prolapsed uterine fibroid, as in our case; this is the first case reported in the literature. The urethrovaginal fistula in our case might have resulted from direct compression of the urethral and vaginal walls between the prolapsed myoma and the pubic bone, leading also to urethral and vaginal ischemia, followed by necrosis and sloughing of the ischemic tissue. This case report reminds us that when a vaginal prolapse of a huge uterine myoma is diagnosed, it should be managed urgently to reduce the risk of fistula formation. In our case, our patient reported late to the hospital (2 days after the beginning of compression). Moreover, management was delayed because the myoma was infected and to avoid the spread of infection, antibiotics were administered prior to the removal of the fibroid.

However, precautions should be taken during transvaginal removal of a huge delivered myoma so as not to aggravate the compression or shearing of adjacent epithelial surfaces. In cases of difficult twisting, morcellation or removal in piecemeal might be advised.

Rarely, an urethrovaginal fistula can also be due to direct trauma of the urethra by a catheter. In this case, during catheterization, the catheter presses the urethral wall against the vaginal wall which is also pressed against the fibroid. This phenomenon may lead to perforation or fragility of the urethral and vaginal walls, or at least to ischemia of the walls, followed by necrosis and sloughing of the ischemic tissue with the occurrence of fistula during the following days. Transurethral bladder catheterization should be performed with caution since urethrovaginal fistula after bladder catheterization has even been observed in the absence of a vaginal mass [4].

## Conclusions

Although urethrovaginal fistula is usually secondary to an intravaginal foreign body, this case report shows that a vaginally prolapsed huge uterine fibroid can also lead to urethrovaginal fistula formation. Therefore, a huge vaginally prolapsed uterine fibroid should be managed urgently in order to reduce the duration of tissue compression and the risk of fistula formation.
